# A Spatial-Temporal Analysis of Cellular Biopolymers on Leaf Blight-Infected Tea Plants Using Confocal Raman Microspectroscopy

**DOI:** 10.3389/fpls.2022.846484

**Published:** 2022-04-18

**Authors:** Alireza Sanaeifar, Dapeng Ye, Xiaoli Li, Liubin Luo, Yu Tang, Yong He

**Affiliations:** ^1^Fujian Colleges and Universities Engineering Research Center of Modern Agricultural Equipment, Fujian Agriculture and Forestry University, Fuzhou, China; ^2^College of Biosystems Engineering and Food Science, Zhejiang University, Hangzhou, China; ^3^Academy of Interdisciplinary Studies, Guangdong Polytechnic Normal University, Guangzhou, China

**Keywords:** leaf blight disease, tea, confocal Raman microspectroscopy, wavelet transform, chemical imaging

## Abstract

The objective of the present study was to characterize the temporal and spatial variation of biopolymers in cells infected by the tea leaf blight using confocal Raman microspectroscopy. We investigated the biopolymers on serial sections of the infection part, and four sections corresponding to different stages of infection were obtained for analysis. Raman spectra extracted from four selected regions (circumscribing the vascular bundle) were analyzed in detail to enable a semi-quantitative comparison of biopolymers on a micron-scale. As the infection progressed, lignin and other phenolic compounds decreased in the vascular bundle, while they increased in both the walls of the bundle sheath cells as well as their intracellular components. The amount of cellulose and other polysaccharides increased in all parts as the infection developed. The variations in the content of lignin and cellulose in different tissues of an individual plant may be part of the reason for the plant’s disease resistance. Through wavelet-based data mining, two-dimensional chemical images of lignin, cellulose and all biopolymers were quantified by integrating the characteristic spectral bands ranging from 1,589 to 1,607 cm^–1^, 1,087 to 1,100 cm^–1^, and 2,980 to 2,995 cm^–1^, respectively. The chemical images were consistent with the results of the semi-quantitative analysis, which indicated that the distribution of lignin in vascular bundle became irregular in sections with severe infection, and a substantial quantity of lignin was detected in the cell wall and inside the bundle sheath cell. In serious infected sections, cellulose was accumulated in vascular bundles and distributed within bundle sheath cells. In addition, the distribution of all biopolymers showed that there was a tylose substance produced within the vascular bundles to prevent the further development of pathogens. Therefore, confocal Raman microspectroscopy can be used as a powerful approach for investigating the temporal and spatial variation of biopolymers within cells. Through this method, we can gain knowledge about a plant’s defense mechanisms against fungal pathogens.

## Introduction

Almost every country in the world consumes tea on a daily basis, making it one of the top three most popular beverages in the world. There are many diseases affecting tea crops, and one of the most serious is tea leaf blight, caused by *Colletotrichum camelliae* Massee (Wang et al., 2016). Typically, anthracnose appears 5–18 days after infection and the affected leaves wither as a result of the damage caused by the development of the lesion (He et al., 2019). Plants that are infected with a pathogen take numerous protective measures to protect themselves. A plant disease resistance can be divided into two categories: organizational structure resistance and chemical resistance. The organizational structure resistance includes the cuticular layers, cork layers, abscission layers, tylose, gum, and so on, while the chemical resistance includes phenolic compounds, phytoalexins, hypersensitive reactions, pathogenesis-related proteins, and so forth (Lu et al., 2018).

In the prevention of fungal diseases, phenolic compounds play a vital role as a kind of chemical resistance. These compounds have the ability to kill pathogens and postinfection productions. The antimicrobial properties of certain phenolic compounds were highlighted recently in a number of studies (Korukluoglu et al., 2008; Mikulic-Petkovsek et al., 2013). Lignin is one of the most significant phenolic compounds. Plant lignins are primarily involved in supporting organs, transmitting sap through lignified parts of the plant’s vascular system, and serving as defensive compounds (Etesami and Jeong, 2018; Soderberg et al., 2021). A reduction in lignin content in crop plants can adversely impact lodging resistance and disease resistance (Pedersen et al., 2005). It has been reported that both abiotic and biotic stresses stimulate some defense genes, including peroxidase, polyphenol oxidase, and phenylalanine aminoamylase, which together are responsible for the formation of lignin within plants (Anand et al., 2009; Shinde et al., 2018). To prevent pathogens from progressing further, lignin forms cork layers around the site of infection. Additionally, tylose is also an important structure found in the infected tissues of the vascular bundle, which consists of hemicellulose, cellulose, and pectin (Pegg et al., 2020; Kashyap et al., 2021). Tylose can affect the vascular bundle by blocking it, thereby preventing infection. Additionally, gum can also provide some resistance to infection by acting on the site of infection (Kashyap et al., 2021). Several studies have demonstrated that biopolymers such as lignin or other phenolic compounds, as well as cellulose or other polysaccharides are adapted to the process of disease resistance in plants. However, the temporal and spatial changes of these biopolymers at the cellular level as a result of infective development are unclear. As a result, further studies are necessary in order to clarify the role of these biopolymers in disease resistance.

There are many conventional chemical analyses that are routinely performed on certain biopolymers, such as high performance liquid chromatography (Mikulic-Petkovsek et al., 2013), but these methods are in most cases invasive and use a large quantity of chemical reagents in order to determine the results (Bellaloui et al., 2012). It should be noted that all of these methods require disintegration of the plant tissues, so only information about composition can be determined, not micromolecular structure and distribution. Also, the distribution of lignin can be determined using an electron microscope (Kiyoto et al., 2018; Polo et al., 2020). The problem is that no domain information can be collected simultaneously, and staining technology must be used in conjunction with microscopes in order to get distribution information. Therefore, a quantitative and qualitative analysis technology should be developed for future research.

Raman microspectroscopy technique has shown a great deal of promise in finding out compositional, structural and spatial information about cellular polymers, due to its high spatial resolution and spectral fingerprint response characteristics (Zhao et al., 2019; Mateu et al., 2020; Saletnik et al., 2021). A remarkable perspective on visualizing cellular walls can be gained by Raman microspectroscopy, which provides detailed information about the physical properties and chemical composition of the cell wall in plants (Pohling et al., 2014). It has also been used to investigate the structure of different types of vascular cells in plants, which are highly complex tissues subject to substantial changes during growth (Jin et al., 2018). This technique has been successful in revealing the spatial and structural characteristics of lignin and cellulose (Ji et al., 2013; Kanbayashi et al., 2019). The Raman peak associated with lignin appears approximately at 1,600 cm^–1^ since the lignin molecule is composed of aromatic ring vibrations which are in symmetry (Gierlinger and Schwanninger, 2006). Furthermore, Raman microspectroscopy can be used to assess changes in lignin composition during plant lignification (Littlejohn et al., 2015), as well as assess differences in lignin distribution and intensity within the walls of different types of xylem cells (Wang et al., 2021). However, there has been no research to our knowledge that has utilized Raman spectroscopy to investigate the time and spatial variations of lignin and cellulose in cells infected with fungal pathogens.

Raman spectroscopy of biological tissues typically produces low-energy signals that are disturbed by noise and fluorescence background. The background-signal contribution is usually reduced by hardware methods at the stage of detection or numerical methods at the stage of data processing (Zeng et al., 2021). However, hardware techniques tend to be inconvenient and costly (Adami and Kiefer, 2013). There are several ways to eliminate background noise, such as direct or modified polynomial fitting and subtraction (Beier and Berger, 2009), rolling-circle spectral filtering (Brandt et al., 2006) and so forth. However, all of these methods of background data reduction are not capable of handling large amounts of data. In order to perform each spectroscopy correction, a special polynomial or circle radius is required, which leads to large mathematical calculations and a disunity calibration reference. Wavelet transform (WT) has been widely used for the denoising and background removal in Raman spectroscopy (Ma et al., 2018; Chi et al., 2019). In general, the WT process is a mathematical algorithm that is capable of localizing signals both on a time and frequency scale. It is also possible for part of the inherent information to be found within a particular sub-space of WT. There is a kind of WT called the discrete wavelet transform (DWT) in which the wavelets are discretely sampled. DWT is a well-established method for improving resolution via spectral denoising and baseline removal (Chen et al., 2011). Researchers in the field of Raman spectroscopy have recently suggested the use of DWT as an ideal strategy for denoising and removing background noise. It has the greatest advantage that bulk data can be processed according to a unified wavelet structure (Tavassoli et al., 2020; Sharan et al., 2021).

It was the objective of the present study to demonstrate the potential of confocal Raman microspectroscopy and the data mining method DWT for the detection of temporal and spatial variation of biopolymers in tea cells induced by leaf blight infection along with the time period of infection for determining the causes of this variation. It is a cutting-edge analytical tool which is capable of imaging lignin and cellulose *in situ*. Our research opens up a novel way of studying plant disease resistance at the cellular scale without involving destruction of the plants.

## Materials and Methods

### Preparation of Tea Samples

We grew tea seedlings [Camellia sinensis (L.) O. Kuntze] of the variety Longjing 43 in pots under natural light, temperature, and manual water conditions for nearly 1 year. In order to confirm that the chosen tea was healthy and free from any fungus infection, the samples were cultivated in a climate incubator (DRX-1200, Hangzhou Runbo Experimental Equipment Co. Ltd., Hangzhou, China) at a fixed temperature (25°C) and humidity (90%) for 10 days. If the plant showed no signs of disease, the plant was chosen for the subsequent infection experiment; otherwise, this procedure was repeated until the plant was healthy. This procedure was followed in order to select healthy tea plants. The fungus (*Colletotrichum camelliae* Massee) was supplied by the Agricultural Experiment Station of Zhejiang University. The mycelium block was inoculated on the tea leaf 4 days after activation. Afterward, the inoculated tea was grown in an incubator for 10 days at a temperature of 28°C and with a relative humidity of 90%.

**FIGURE 1:**
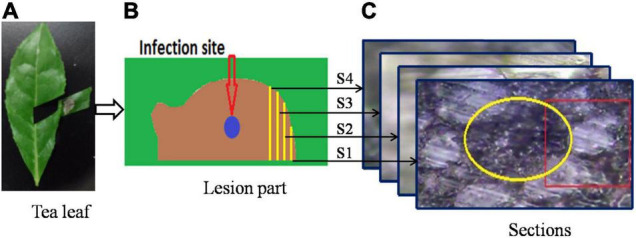
A schematic of the sample preparation process: **(A)** infected tea leaf, **(B)** enlargement of infected area, and **(C)** bright field microscopy images of sections.

The lesion part (Figure 1B) was then cut from the tea leaf (Figure 1A), and prepared for resin embedding in accordance with the following steps. (1) Double fixation: first, the specimen was fixed with 2.5% glutaraldehyde in phosphate buffer (pH 7.0) for more than 4 h, and then it was washed three times in the phosphate buffer after it had been fixed; finally, the specimen was postfixed with 1% OsO4 in phosphate buffer (pH 7.0) for an hour. (2) Dehydration: a series of increasing concentrations of ethanol was used to dehydrate the specimen (50, 70, 80, 90, 95, and 100%). About 15–20 min were spent in each step, then the solution was transferred to absolute acetone. (3) Infiltration: in a 1:1 mixture of absolute acetone and the final spurr resin mixture, the specimen was dissolved for 1 hour at room temperature. Afterward, the mixture was treated with acetone-resin (1:3) for 3 h and left overnight. (4) Embedding and semithin sectioning: in capsules containing embedding medium, specimens were heated at 70°C for approximately 9 h. A microtome (Thermo Fisher Finesse 325 paraffin) was used to cut sections of 5 μm thick without further processing. The cutting direction was edge toward the infection site. Four sections were selected for Raman spectroscopy, and each section was separated by about 50 μm. Figure 1 illustrates the process of sample preparation. The schematic diagram of the infected part and the locations of four sections are shown in Figure 1B. The infection site was at the center of the lesion part (represented with a blue circle in Figure 1B). The sectioning was done from the edge to the center. Accordingly, S1 was designated as the first section, namely the shortest infection time and the slightest extent of infection. The second, third, and fourth sections were labeled S2, S3, and S4, respectively. Within these four sections, S4 had the longest infection period and the most serious degree of infection.

### Spectroscopy Acquisition

A confocal Raman microspectrometer (Renishaw, United Kingdom/Via-Reflex 532/XYZ) equipped with a diode-pumped solid state laser (50 mW at 532 nm) was applied to collect the spectra. The use of 532 nm wavelength excitation to analyze cellulose and lignin contributions has demonstrated that the signal from these biomolecules can be used for detailed analysis of the cell wall, despite the non-bleaching selectivity. The comparative analysis of NIR (785 nm)-excited versus 532 nm-excited Raman spectra of the same samples indicated that imaging and characterization of cellular walls may yield greater advantages from high scattering at the visible wavelength, rather than from low bleaching at the NIR wavelength (Heiner et al., 2018; Zeise et al., 2018).

The laser power applied to the sample was 0.5 mW, and the incident laser beam was focused onto the sample surface with a 50× objective lens at 0.75 numerical aperture. During the mapping process, an integration time of 10 s and steps of 1 μm were assigned, and every pixel was represented by a single scan. The four sections were scanned near the vascular bundle, which can be seen in Figure 1C, where the yellow circle represents the vascular bundle and the red rectangle represents the scanning area. The Raman spectrometer was configured to map the Raman spectral data with a spatial resolution of 1 μm in both horizontal and vertical directions. Due to the differences in tissue types, the scanning extent varied for the different sections, with 1,053, 735, 936, and 780 points for the S1, S2, S3, and S4 sections, respectively. For the purpose of removing background, a Raman spectroscopy of pure spurr resin was also performed.

### Discrete Wavelet Transform

**FIGURE 2:**
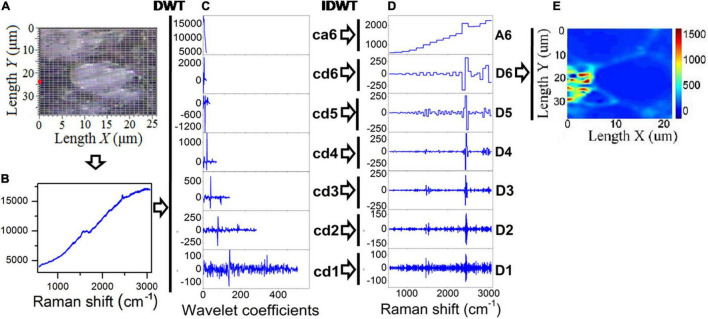
DWT and IDWT data processing of Raman spectra. **(A)** Optical microscope image of tea tissue, **(B)** Raman spectroscopy of a sampling point, **(C)** wavelet decomposition coefficients, **(D)** wavelet reconstruction signals, and **(E)** chemical image generated from the reconstruction signal (D6).

As explained in the introduction, DWT provides an extremely useful method to remove Raman background information. In wavelet analysis, signals are decomposed into discrete levels of resolution, which is known as multi-resolution. Due to the fact that the background consists primarily of low-frequency features, this background is removed from the spectrum. To denoise a specific Raman signal, in addition to the DWT for decomposition (analysis), an inverse DWT (IDWT) is also applied for reconstruction (synthesis; Li et al., 2020). Sub-band filters can be used to decompose and reconstruct wavelets. A schematic view of a WT used in the present study for minimizing the effects of fluorescence in Raman chemical imaging is shown in Figure 2. Figure 2A illustrates the Raman spectral scanning region of healthy tea tissue, where each grid point represents a sampling point. Figure 2B shows a Raman spectral response curve for a sample circled with a red line in Figure 2A, and this sample is taken as an example to illustrate the inhibition of fluorescence by WT. The Daubechies 1 wavelet was adopted for decomposition and reconstruction in this study, and then seven wavelet decomposition coefficients were calculated as a6, d6, d5, d4, d3, d2, and d1, as seen in Figure 2C. Following this, seven wavelet reconstruction signals (A6, D6, D5, D4, D3, D2, and D1) were computed by IDWT from the corresponding wavelet decomposition coefficients, as shown in Figure 2D. As illustrated in Figure 2E, the Raman chemical image can finally be derived from the wavelet reconstructed signal (D6) for all sampling points.

The optimization approach for chemical imaging was implemented in three steps in this study. The first step involved wavelet decomposition of the signal, followed by wavelet reconstruction, and the third step involved integrating the biopolymer feature bands based on the reconstruction structure D6.

## Results

### Raman Spectrum of Leaf Blight-Infected Tea Tissue

**FIGURE 3:**
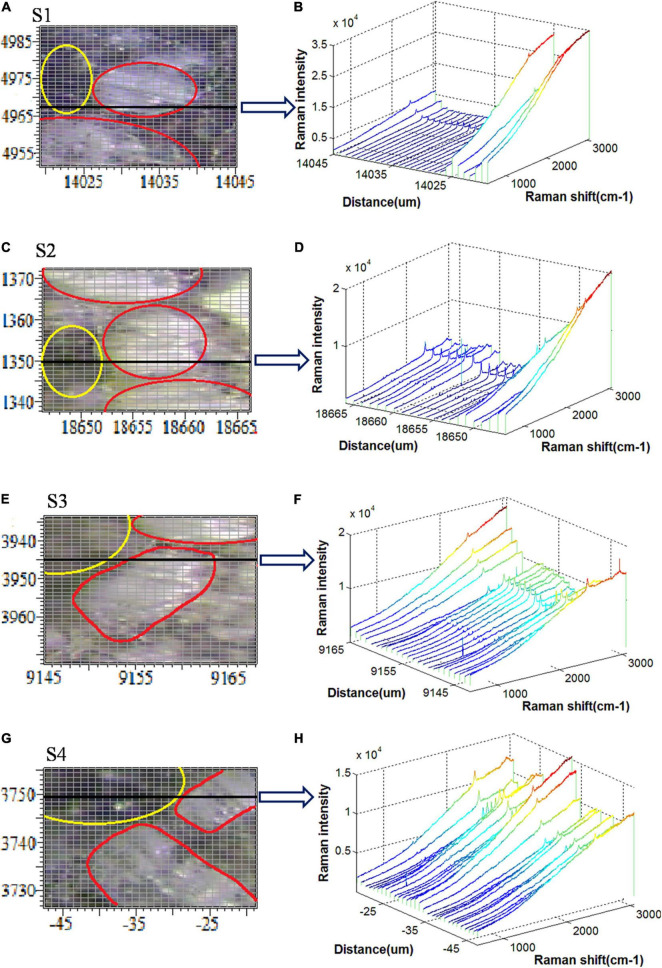
Raman spectral scanning regions and spectral responses for **(A,B)** S1, **(C,D)** S2, **(E,F)** S3, and **(G,H)** S4.

Raman spectral scanning includes the vascular system, and vascular bundles are tightly surrounded with bundle sheath cells as shown in Figure 1C. The details of the scanning regions of the four sections and their typical Raman spectral responses are shown in Figure 3.

Figures 3A,C,E,G show the bright-field microscopy images of scanning areas of the S1, S2, S3, and S4, respectively, the tissues within the yellow ellipse represent the vascular bundles, whereas the tissues within the red ellipse represent the bundle sheath cells. Also, Figures 3B,D,F,H show the Raman spectra of the sampling points along the horizontal black line in each scanning area. It is important to note that these black lines traverse both vascular bundles and bundle sheath cells. According to Figure 3B, the Raman intensity of the vascular bundle lying on the black line is much larger, up to 3.5 × 10^4^. However, the Raman intensities of the other sampling points are almost equal to zero. Accordingly, it is concluded that there is a substantial difference between the Raman spectral response of vascular bundle and bundle sheath cells in the S1 section. The Raman spectral intensity of the vascular bundle is still higher than that of the bundle sheath cell in the S2 section, although the difference is less than that of the S1 section (Figure 3D). The Raman spectral intensity of the vascular bundle is shown in Figure 3F to be greatly reduced, while the Raman spectral intensity of the bundle sheath cell is highly increased, and the difference between the two is further reduced in the S3 section. As shown in Figure 3H, the Raman spectral intensities in the bundle sheath cell and the vascular bundle are comparable in the S4 section. Generally, the Raman intensity of the vascular bundles in the four sections is relatively high, which may be attributed to the high lignin content of the vascular bundles (Richter et al., 2011). Also, the Raman intensities of vascular bundles are ranked in descending order as S1, S2, S3, and S4 indicating that the lignin and other biopolymers decreased from S1 to S4 along with an increase in infection severity. In addition, it is noteworthy that the Raman spectral response differences of the vascular bundle and bundle sheath cells gradually decrease from S1 to S4, indicating that the differences in structure and composition between these two types of tissues are gradually diminishing as the disease severity increases. It may be because the pathogen destroys the ordered structure of these tissues and decomposes many of the biopolymers that compose their cell walls.

### Principal Component Analysis-Based Temporal Classification of Infection

An overview of the temporal and spatial comparisons of four sections was presented in the previous section. In order to have a detailed temporal analysis of these sections, Raman spectra of the same tissue in four different sections were selected. There are two parts to the bundle sheath cell, namely, the cell wall and the inside of the cell. As the proportion of vascular bundles was small in the scanning region, the analysis was conducted on the whole bundle. In this regard, three different parts were distinguished: the cell wall of the bundle sheath cell, the inside of the bundle sheath cell, and the vascular bundle. As shown in Figure 3, 60 points were recorded for each part. Sample points for the vascular bundle were selected within the yellow circles, samples for the cell wall were selected along the edge of the red lines, and samples for intracellular were selected within the red areas.

**FIGURE 4:**
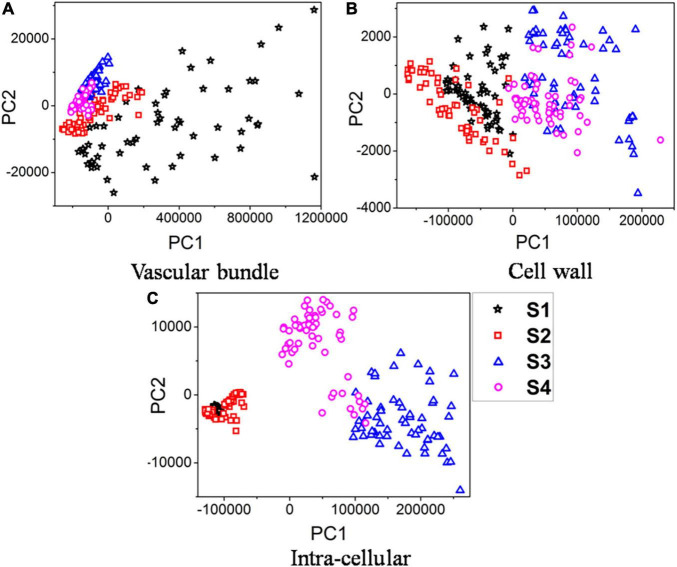
PCA plots of samples infected over time. **(A)** Vascular bundle. **(B)** Cell wall. **(C)** Intra-cellular.

To simplify the data visualization process, principal component analysis (PCA) was first used to re-express the original Raman signals. In three different parts, 240 Raman spectral samples in sections S1, S2, S3, and S4 were analyzed using PCA. The PCA was applied to the Raman spectral region of 579–3,062 cm^–1^. Figures 4A–C illustrate the results of PCA analyses for the first two principal components (PC1, PC2) of the vascular bundle, cell wall, and intracellular components, respectively.

Results indicated that the first two components, in three separate parts, contained over 99% of the data variance in classification based on four different stages of infection. Figure 4A indicates that the vascular bundle signal in S1 is quite different from that in the other three sections, with a more scattered distribution. The reason is that the Raman spectra of vascular bundles in S1 were significantly higher than those of S2, S3, and S4 (see Figure 3). Additionally, since the vascular bundle itself is composed of xylem and phloem, these 60 spectra are scattered throughout due to the fact that the vascular bundle itself is composed of various structures and substances. However, the vascular bundle spectra of S2, S3, and S4 were more concentrated, indicating that the structures of the bundle were not greatly different. It is possible that the structure has been damaged in these sections. This conclusion is in accordance with the results in the previous section. According to Figure 4B, the boundary between these four sections was not clearly defined, there were some overlaps. Specifically, the S1 overlapped the S2 and the S3 overlapped the S4. However, the boundary of S1 and S2 was clearly distinct from S3 and S4, indicating the composition of the cell wall in S3 and S4 was clearly different from S1 and S2. Also, in Figure 4C, the boundary was clearly defined, except in S1 and S2. The analysis revealed the components of S1 and S2 inside the cell were similar, but they differed greatly in S3 and S4. In spite of overlaps in the PCA score plots, the clustering results were satisfactory. Thus, the extracted two principal components were able to reveal Raman spectral features in the vascular bundle, cell wall, and intracellular structures of the four sections, ensuring effective identification. Furthermore, the principal component distribution indicates that Raman spectroscopy can reflect differences in four sections, i.e., Raman spectroscopy is capable of recognizing four sections.

### Analysis of Raman Spectra Based on Characteristic Peaks

**FIGURE 5:**
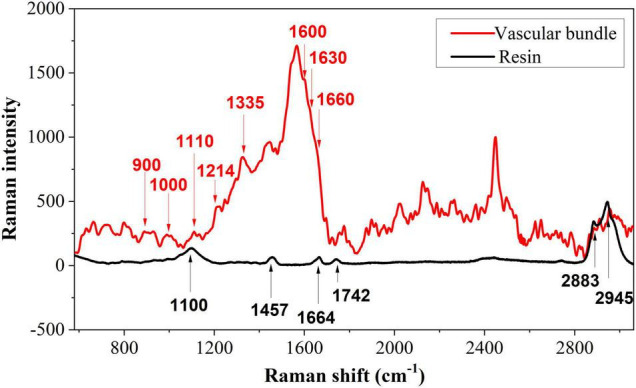
A typical Raman spectrum of the vascular tissue and the spurr resin.

It is essential to determine the characteristic peaks of the background before analyzing the distribution of biopolymer in tea cells. Due to the presence of spurr resin in a semi-thin transverse section of the tea tissue, background disturbance from the spurr resin must be removed. Figure 5 shows representative Raman spectra of vascular tissue and its background. The spectrum of the vascular tissue is randomly selected from the vascular bundle on the first section in Figure 3A marked with a yellow circle, while the spectrum of the background comes from the pure spurr resin. For removing fluorescence interference and highlighting the signal, polynomial fitting and subtracting were employed. There were six main peaks in the resin, which could interfere with the analysis of the sample. Especially the peak at 1,664 cm^–1^, which is also included in the sample. The spectroscopy of the vascular bundle generated a strong and broad peak at 1,600 cm^–1^. This peak may be comprised of four peaks: 1,570, 1,600, 1,630, and 1,660 cm^–1^. These last three peaks were related to lignin. The 1,600 cm^–1^ was assigned to aromatic ring mode, the 1,630 cm^–1^ to ring conjugated C=C stretching of coniferaldehyde, and the 1,660 cm^–1^ to ring conjugated C=C stretching of coniferyl alcohol (Hänninen et al., 2011; Mateu et al., 2020). It is noteworthy that these three peaks appeared in all phenolic compounds. There was also a peak at 1,214 cm^–1^ corresponding to aryl-O of aryl-OH and aryl-O-CH_3_ and guaiacyl ring mode (with C=O group) that was related to lignin. The peak at 900 cm^–1^ was associated with bending of HCC and HCO at C6, and the signal around 1,000 cm^–1^ was associated with heavy atom stretching (CC and CO; Adapa et al., 2009). The two peaks were related to cellulose. The peak at 1,110 cm^–1^ may consist of two peaks (1,095 and 1,123 cm^–1^), which were related to cellulose as well. Additionally, these peaks corresponded to the highest polysaccharide levels. Signals at 1,335 cm^–1^ were caused by HCC and HCO bending or by aliphatic O–H bending. Both lignin and cellulose showed signals at this wavelength (Adapa et al., 2009). Since the resin possesses a peak at 1,660 cm^–1^ as well, it was selected as a standard peak for semi-quantitative comparisons. It should also be mentioned that the sharp peak around 2,400 cm^–1^ that was caused by noise has been removed as it represents a spurious peak from the spectrometer.

Following this, biopolymer analysis was conducted on three positions, namely the vascular bundle, the cell wall, and the intracellular. According to the results of the PCA, Raman spectroscopy can demonstrate differences in the four sections. Raman spectroscopy was averaged to simplify the analysis, and then polynomial fitting and subtracting were implemented to eliminate fluorescence interference and highlight the signal.

**FIGURE 6:**
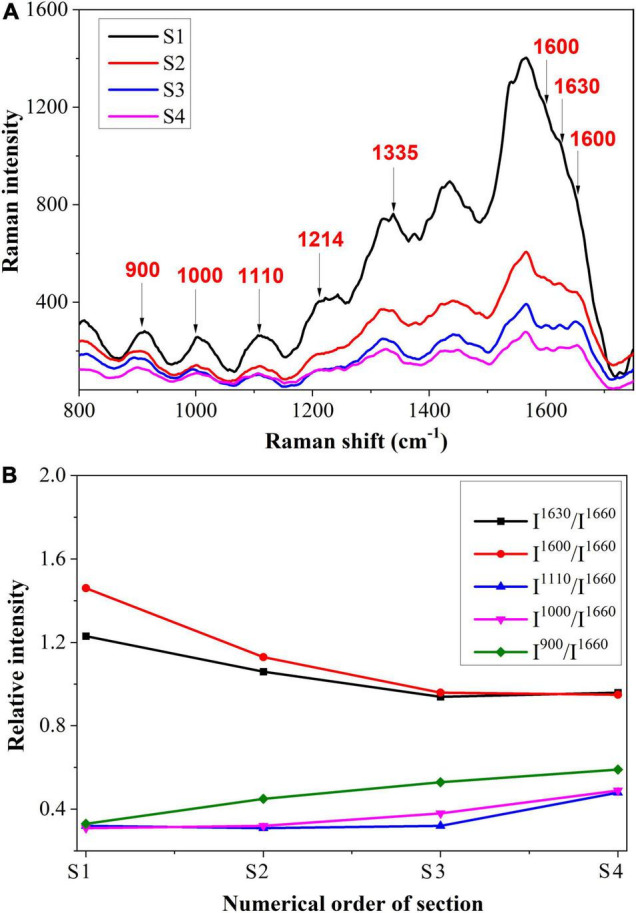
**(A)** Raman spectroscopy and **(B)** Semi-quantitative intensity analysis of vascular bundle.

Figure 6A shows the spectrum of the vascular bundle from S1 to S4. In Figure 6B, a semi-quantitative analysis was performed and a standard peak at 1,660 cm^–1^ was chosen as a baseline in order to make an accurate comparison. From S1 to S4, the Raman intensity of 1,600 and 1,630 cm^–1^ that corresponded to lignin decreased. This finding revealed that the more severe infection within the vascular bundle resulted in less lignin content. In this case, it may be because the structure of the vascular bundle was damaged by the pathogen, causing the lignin to be distributed irregularly. A small amount of lignin might be expelled from the vascular bundle; therefore, the content of lignin in the vascular bundle was reduced. The decomposition of lignin caused by fungi is another cause for the reduction of lignin. Research has previously revealed that certain fungi have the capacity to decompose lignin (Wei, 2012). In contrast, the peaks relating to cellulose, such as 900 and 1,000 cm^–1^, were increased from S1 to S4. In other words, the maximum cellulose content was located in the most serious section. This was due to the fact that, when a pathogen invades the vascular bundle, the xylem will produce a substance called tylose, consisting of cellulose, hemicellulose, and pectin. The vascular bundle was damaged by the pathogen, thus the defense structure tylose was produced to block the vessels and prevent the spread of the invasion.

**FIGURE 7:**
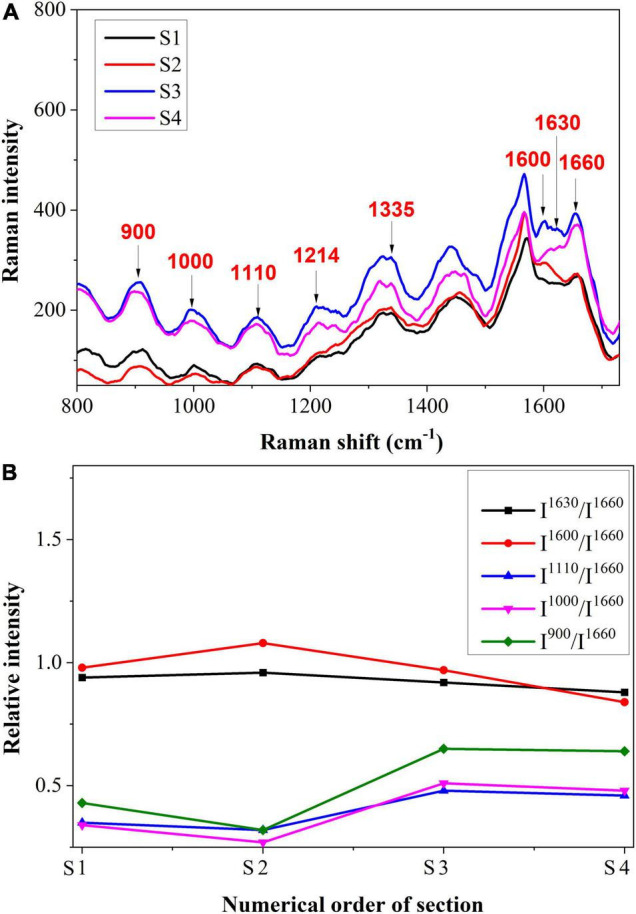
**(A)** Raman spectroscopy and **(B)** Semi-quantitative intensity analysis of cell wall.

The spectrum of the cell wall from S1 to S4 is shown in Figure 7A, and the semi-quantitative analysis is shown in Figure 7B. It was shown that the Raman intensities of 1,630 and 1,600 cm^–1^ increased first and then decreased slightly, which was different from the variation trend in vascular bundles. This may be due to the plant’s inherent resistance to stress. To avoid further damage, the plant may increase its lignin content (Chérif et al., 1991). The Raman intensities of cellulose were initially decreased and then increased. The reduction of cellulose in cell walls may be a result of pathogen decomposition. In S3 and S4, however, the content increased, which may be due to the emergence of some new substances. In previous research, it was reported that hydroxylproline-rich glycolproteins are present in cell walls to protect them against pathogens (Deepak et al., 2008).

**FIGURE 8:**
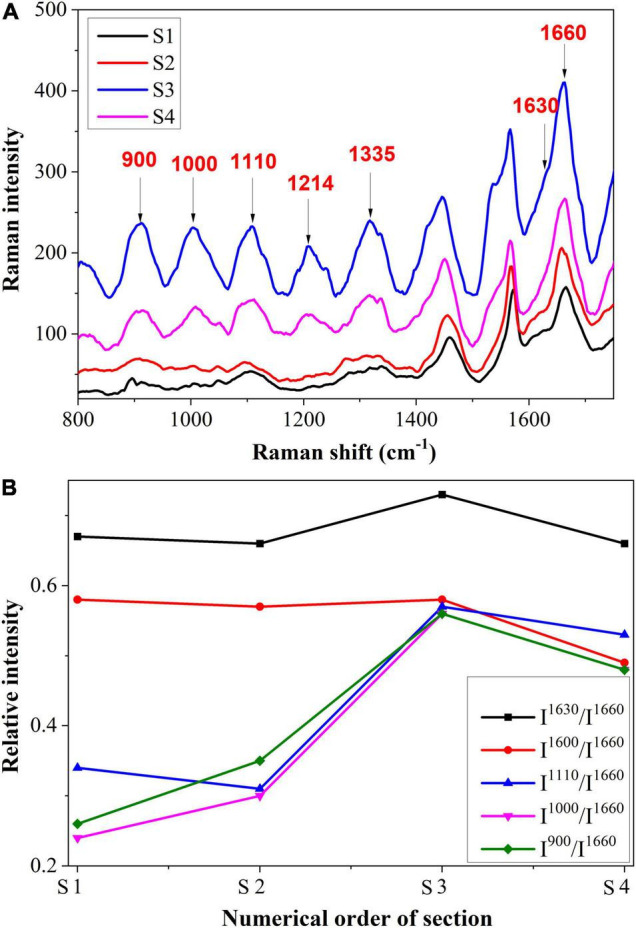
**(A)** Raman spectroscopy and **(B)** Semi-quantitative intensity analysis of intra-cellular.

In Figure 8A, we present the intracellular spectrum from S1 to S4, and Figure 8B shows the semi-quantitative analysis. As can be observed, there was a similar variation trend in Raman intensity between the intracellular and the cell wall. There was an increase in intensity at 1,600 and 1,630 cm^–1^, followed by a decrease. As the Raman shifts at these two peaks are observed in all phenolic compounds, this may indicate that the amount of phenolic compounds within the cell was increased. In addition to their role in the prevention of fungal diseases, phenolic compounds also exert toxic effects on pathogens and their postinfection activities (Korukluoglu et al., 2008; Mikulic-Petkovsek et al., 2013). Thus, the presence of phenolic compounds also contributed to the plant’s active resistance. In addition, the Raman bands at 900, 1,000, and 1,110 cm^–1^ increased first and then decreased. This may be due to another organizational structure resistance, namely gum, which contains substances such as cellulose, semi-cellulose, lignin, etc. Gum also functions to prevent pathogens from spreading further (Kashyap et al., 2021).

### Raman Chemical Imaging of Biopolymers

**FIGURE 9:**
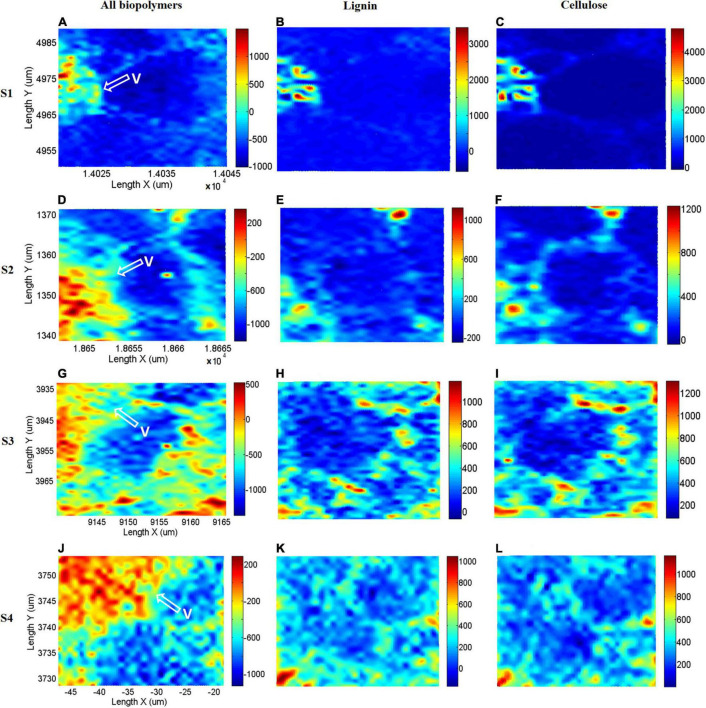
Chemical images of four sections: S1 **(A–C)**, S2 **(D–F)**, S3 **(G–I)**, and S4 **(J–L)**. **(A,D,G,J)**: all polymers band intensity, **(B,E,H,K)**: lignin band intensity, and **(C,F,I,L)**: cellulose band intensity.

As the scanning regions contain thousands of Raman spectra, DWT was applied to remove the noise and fluorescence interference from the spectra. The chemical images of lignin and cellulose were reconstructed using DWT. The lignin band of 1,600 cm^–1^ which was assigned to aromatic ring mode was used to establish chemical images. In order to visualize the chemical image of lignin, spectral intensity from 1,589 to 1,607 cm^–1^ was integrated. Furthermore, the chemical image of cellulose was produced with a range of intensities from 1,087 to 1,100 cm^–1^. Also, the CH-stretching region between 2,980 and 2,995 cm^–1^, which was assigned to all polymers, such as cellulose, hemicellulose, pectin and lignin, was used to establish chemical images (Gierlinger and Schwanninger, 2006). The chemical images of the four sections are illustrated in Figure 9, and the white arrowheads indicate the vascular bundles. The distribution of lignin in the slightest infected area is almost limited to the vascular bundles and the corner of the bundle sheath cell wall (Figure 9B). As shown in Figure 9C, there is a distribution of cellulose in the vascular bundle as well as in the entire bundle sheath cell wall. Additionally, the distribution area of all biopolymers (Figure 9A) was greater than that of lignin and cellulose due to the presence of hemicellulose and pectin. As the extent of infection was increased in Figure 9E, the distribution of lignin in the vascular bundle became irregular. In addition to the corner of the bundle sheath cell, lignin is produced throughout the entire cell wall. Figure 9F shows that the distribution of cellulose was similar to that of lignin. The distribution of all biopolymers (Figure 9D) revealed that there was an accumulation in the vascular bundle. It is shown in Figure 9H that most lignin is distributed in the cell wall, and the structure of the vascular bundle is damaged, therefore the amount of lignin in the bundle is reduced as compared to Figures 9B,E. In addition, an abundance of lignin or phenolic compounds accumulated inside the cell. The distribution of cellulose (Figure 9I) was also extended to the intracellular space. As shown in Figure 9G, the photochemical image of biopolymers shows cellulose, hemicellulose, pectin, and lignin are abundantly produced. Lastly, in the most seriously infected section, lignin or other phenolic compounds (Figure 9K), as well as cellulose (Figure 9L) were scattered throughout the scanning region. Moreover, a significant amount of biopolymers were accumulated (Figure 9J) in the vascular bundle, which was associated with the formation of tylose.

## Discussion

While it is known that lignin, phenolic compounds, tylose, and other biopolymers contribute substantially to plant disease resistance, their temporal and spatial variations with the development of infection have not been well investigated. Confocal Raman microscopy was used in this study for the analysis of biopolymers on a micrometer scale. There was a clear visualization of the spatial distribution of the lignin. Using semi-quantitative analysis, it was found that the Raman intensities of lignin decrease gradually from S1 to S4, while cell wall and intracellular Raman intensities increase. There was also an increase in cellulose content in the vascular bundle as well as in the cell wall and intracellular spaces. This may be due to the fact that in the vascular bundle, there is primarily resistance provided by hemicellulose, cellulose or pectin, specifically by tylose. In the bundle sheath cell, both biopolymers contribute to the resistance factor. There was a rising trend in both Figures 7B, 8B. Variations can be caused by antimicrobial polymers such as phenolic compounds, hydroxyproline-rich glycoproteins, gum, etc. It should be noted that the semi-quantitative analysis has confirmed the chemical images. The lignin distribution in the vascular bundle has become increasingly irregular from S1 to S4. In this case, the pathogens have caused damage to the structure of the vascular bundle, which has led to a loss of rigidity in the vascular, eventually degenerating and collapsing. The lignin content of the vascular bundle was reduced as a result. Using the CH-stretching region between 2,980 and 2,995 cm^–1^, the chemical image of all biopolymers containing hemicellulose, cellulose, and pectin determined that these polymers were gradually accumulated in the vascular bundle. In addition, the distribution of lignin in bundle sheath cells demonstrated that in sections where the infection was severe, like S3 or S4, the cells were thick with lignin or phenolic compounds in order to resist pathogen invasion. These results are consistent with previous research. Researchers discovered that phenolic compounds show defense responses characterized by rapid and early accumulation at the infection site, which prevents the pathogen from spreading (Chérif et al., 1991). The formation of lignin was one of the responses to these conditions (Zhao et al., 2019; Mateu et al., 2020; Saletnik et al., 2021). In bundle sheath cells, cellulose was distributed similarly to lignin. This occurred due to the synthesis of various polysaccharides to combat the pathogen, such as hydroxyproline-rich glycoproteins.

It has been shown in our study that the wavelet transform can be highly efficient in reducing low-frequency fluorescence signals that accompanied and interfered with the Raman spectra of plant tissues. This finding was also consistent with other studies that have shown that the wavelet transform eliminates the low-frequency fluorescence signal. Using wavelet coefficients at level 6, the Raman spectral difference between untreated samples and alkali-treated samples of rice straw was quantitatively assessed after residual noise and fluorescent background was removed (Li et al., 2020). In support of our findings, researchers have indicated that spectroscopic methods combined with DWT analysis offered a rapid and non-destructive approach for estimating integral chemical information (Wang et al., 2018; Yao et al., 2018; Li et al., 2019; Liu et al., 2021). It was possible to perform wavelet transform on spectra using a formula which involves manipulating and adjusting the wavelet function to generate values that distinguish various frequencies. DWT analysis was therefore able to access a larger amount of information embedded within the plant spectrum resulting in an advanced ability to extract information from it.

Although imaging techniques are widely used to analyze cell walls, a thorough understanding of their structure is still lacking. The classic methods for imaging cell walls are to stain or label their chemical components (e.g., using immunolabeling); however, such analyses rely on the results of a large number of mixed samples obtained from enzyme-isolated or pulverized plant tissues (Zhao et al., 2019). Imaging of plant cell walls is performed using classical methods to determine the spatial and temporal changes in polysaccharides within cell walls during the growth process (Hervé et al., 2011; Tobimatsu et al., 2013). However, these techniques have limitations due to their expense, depth of penetration and spatial resolution, and some of them use fluorescent imaging, which poses challenges due to photobleaching and genetic modifications. In addition, these microscopy approaches are non-quantitative for analyzing cell walls. Confocal Raman microspectroscopy, which does not require stains or fluorescent indicators to operate, has greatly improved label-free imaging and enabled more in-depth and detailed study of the plant cell wall. In addition to measuring cell wall components simultaneously, it also allows observing changes in cell wall morphology (Wu et al., 2016).

It should be noted that this paper has some limitations that can be improved in future research. Increasing the number of sections in this article would provide a comprehensive description of the entire infection process. Furthermore, the scanning area can be large enough to allow for the representation of the entire cell. It is demonstrated in this paper that a novel approach is used to examine the mechanism of plant resistance, which contributes to a better comprehension of biopolymer variations in the process of fungal infection. This is the first time that confocal Raman microspectroscopy has been applied to study the time and spatial variation of damage to the cell wall in fungal-host interactions. The method proposed in this paper is potentially applicable for the *in situ* quantification of polymers in the cell wall structure as well as composition. Researchers can evaluate the damage to the cell walls directly from epidermal tissue using Raman microspectroscopy in the future with the help of laser penetration of microdots infected by pathogens. It has been demonstrated that the microspectroscopy method can provide real-time monitoring of the structure of the poplar cell wall during the process of ethyl-3-methylimidazolium acetate solubilization (Zhang et al., 2014). Accordingly, in this non-destructive *in situ* method, there is no requirement for samples to be cut or significantly altered in order to be measured, thus preserving the integrity of samples.

## Conclusion

This study used confocal Raman microspectroscopy for the first time to further investigate the temporal and spatial variations of biopolymers observed in tea leaf blight-infected cells. We analyzed the Raman spectra of four sections that show progression of infection in order to conduct a semi-quantitative Raman intensity analysis of biopolymers. PCA was able to reveal Raman spectral signatures in the vascular bundle, cell wall, and intracellular structures of each of the four sections, which enabled Raman spectroscopy to be used to differentiate between the four sections. A semi-quantitative analysis revealed that the Raman intensities of lignin gradually declined as infection progressed, whereas those of the cell wall and intracellular regions increased. As well, cellulose quantities increased in three parts with infection severity. It was proposed that the wavelet transform could be used for *in situ* Raman chemical imaging. With its excellent multiscale analysis capability, wavelet transform was able to exclude low-frequency fluorescence interference as well as high-frequency cosmic rays from the image. It was found that the two-dimensional chemical images of lignin, cellulose, and all biopolymers are analyzed via wavelet-based data mining based on the acquisition of the characteristic wavelengths ranging from 1,589 to 1,607 cm^–1^, 1,087 to 1,100 cm^–1^, and 2,980 to 2,995 cm^–1^, respectively. The semi-quantitative analysis results were completely consistent with the chemical images that were obtained. Therefore, confocal Raman microspectroscopy can be considered a powerful tool that can be used to analyze cellular biopolymers to identify changes occurring due to fungal infection.

## Data Availability Statement

The raw data supporting the conclusions of this article will be made available by the authors, without undue reservation.

## Author Contributions

AS: writing—original draft, investigation, conceptualization, visualization, formal analysis, and software. DY: methodology and funding acquisition. XL: conceptualization, validation, writing—eview and editing, supervision, project administration, and funding acquisition. LL: resources and data curation. YT: data curation. YH: writing—review and editing, supervision, project administration, and funding acquisition. All authors contributed to the article and approved the submitted version.

## Conflict of Interest

The authors declare that the research was conducted in the absence of any commercial or financial relationships that could be construed as a potential conflict of interest.

## Publisher’s Note

All claims expressed in this article are solely those of the authors and do not necessarily represent those of their affiliated organizations, or those of the publisher, the editors and the reviewers. Any product that may be evaluated in this article, or claim that may be made by its manufacturer, is not guaranteed or endorsed by the publisher.
